# Brain metabolic alterations in individuals with chronic non-specific neck pain assessed using proton magnetic resonance spectroscopy

**DOI:** 10.1038/s41598-025-24339-3

**Published:** 2025-11-18

**Authors:** Rungtawan Chaikla, Suchart Kothan, Marco Barbero, Deborah Falla, Munlika Sremakaew, Sureeporn Uthaikhup

**Affiliations:** 1 Department of Physical Therapy, Integrated Neuro-Musculoskeletal, Chronic Disease, and Aging Research Engagement Center (ICARE), Faculty of Associated Medical Sciences, Chiang Mai University, Chiang Mai, 50200 Thailand; 2 Center of Radiation Research and Medical Imaging, Department of Radiologic Technology, Faculty of Associated Medical Sciences, Chiang Mai University, Chiang Mai, Thailand; 3 Department of Business Economics, Health and Social Care, Rehabilitation Research Laboratory 2rLab, University of Applied Sciences and Arts of Southern Switzerland, Manno, Switzerland; 4 Centre of Precision Rehabilitation for Spinal Pain (CPR Spine), School of Sport, Exercise and Rehabilitation Sciences, College of Life and Environmental Sciences, University of Birmingham, Birmingham, UK

**Keywords:** Brain metabolites, Chronic pain, Magnetic resonance spectroscopy, Neck pain, Non-communicable diseases (NCDs), Pain outcomes, Brain imaging, Magnetic resonance imaging, Chronic pain

## Abstract

Altered brain metabolites in pain-related regions provide insights into the underlying mechanisms of chronic pain. However, brain metabolites alterations in chronic non-specific neck pain remain unknown. This study aimed to investigate brain metabolite concentrations in individuals with chronic non-specific neck pain and their relationships with pain-related outcomes. Participants included 30 individuals with chronic non-specific neck pain and 30 pain-free controls. Absolute concentrations and metabolite ratios of total creatine (tCr), choline (Cho), myo-inositol (mI), N-acetylaspartate (NAA) and glutamate/glutamine (Glx) were measured in regions involved in pain processing and modulation, including dorsolateral prefrontal cortex (DLPFC), primary somatosensory cortex (S1), insula and thalamus, using proton magnetic resonance spectroscopy (^1^H-MRS). Compared to controls, participants with neck pain exhibited decreased mI and mI/tCr (left DLPFC and thalamus), NAA and NAA/tCr (right S1) and Glx and Glx/tCr (right DLPFC) and increased Cho and Cho/tCr (left S1) (adjusted p-values < 0.05). Altered metabolite levels were correlated with pain duration, intensity, extent, disability and PPT at C2-3 (r ranged from − 0.48 to 0.55, adjusted p-values < 0.05). The results suggest that ¹H-MRS detects altered levels of mI, NAA, Glx and Cho in specific brain regions involved in pain regulation, which may contribute to the persistence of neck pain.

## Introduction

Non-specific neck pain is defined as pain without an unidentified pathoanatomical cause^1^. It often progresses to chronicity, persisting for more than 3 months^1^ and is associated with increased disability and reduced quality of life^2^. Individuals experiencing chronic non-specific neck pain exhibit a range of clinical features, including variations in the subjective report of their pain characteristics (e.g., intensity, extent and disability), measures of pain sensitivity, psychological distress and reports of functional ability^3,4^. However, complaints of pain or self-reported disability are inherently subjective and may not always correspond with objective physical findings. As such, examining physiological indicators, such as neurochemical changes in the brain may help identify mechanisms of chronic neck pain and thereby provide further insight into the persistence of pain and disability.

Proton magnetic resonance spectroscopy (^1^H-MRS) is a non-invasive technique that quantifies the concentrations of various neurochemicals in brain tissue^5^. ^1^H-MRS generates a spectrum to assess neurometabolites, which provide valuable insights into brain health (i.e., neuronal integrity, energy metabolism, neurotransmitter function and inflammatory processes)^6–8^. Imaging studies have shown that changes in brain metabolites, including *N*-acetylaspartate (NAA), creatine (Cr), choline (Cho), myo-inositol (mI) and glutamate/glutamine (Glx) are associated with pain processing in various chronic pain conditions^7,9,10^. In general, NAA is regarded as a marker of the density and mitochondrial function^7^; Cr reflects the brain’s energy metabolism^6^; Cho is associated with membrane turnover and neuroinflammation^6^; mI indicates neuroinflammatory and glial activation^8^; and Glx represents excitatory and inhibitory neurotransmitters involved in pain signaling and central sensitization^7^. The brain regions commonly involved included the dorsolateral prefrontal cortex (DLPFC), primary somatosensory cortex (S1), anterior cingulate cortex (ACC), insula and thalamus^7,9,10^.

A previous study demonstrated that patients with non-specific neck pain exhibited changes in regional cerebral blood flow, including decreased perfusion near the hippocampus and increased perfusion in the left insula, patterns which were not observed in those with neck pain resulting from a whiplash trauma^11^. A recent study also reported differences in the metabolite concentration and relative glutamate levels in the ACC and total Cho levels in the DLPFC among patients with chronic whiplash-associated disorders (WAD) presenting with neuropathic pain, compared to those with WAD without neuropathic pain and healthy controls^12^. Furthermore, relative glutamate levels in the ACC as well as relative NAA and total Cho levels in the DLPFC could predict the presence of neuropathic pain in patients with chronic WAD.

Alterations in neurometabolites have been suggested to play an important role in the development or persistence of chronic pain^7,9,10^. However, specific metabolite changes in individuals with chronic non-specific neck pain remain unexplored. Investigating these alterations may provide valuable insights into central pain modulation and the underlying mechanisms, ultimately supporting the development of more targeted and effective treatment strategies tailored to individual pain profiles. The aim of this study was to investigate the concentrations of brain metabolites in regions integral to pain processing networks (i.e., DLPFC, insula, S1 and thalamus), using ^1^H-MRS in individuals with chronic non-specific neck pain compared to pain-free controls. The study also explored relationships between levels of brain metabolites and self-reported pain outcomes and measures of pain sensitivity. It was hypothesized that individuals with chronic non-specific neck pain would exhibit altered concentrations of brain metabolites in regions integral to pain processing networks.

## Methods

### Study design and participants

This cross-sectional study was conducted as part of a larger research project (grant number FRB650031/0162 and FRB660046/0162). The period of recruitment was between January and November 2023, with data collection conducted at the research unit of the Departments of Physical Therapy and Radiologic Technology, Chiang Mai University. As there has been no previous research examining neurochemical metabolite concentrations in patients with chronic non-specific neck pain, the sample size for the present study was estimated based on the study design. Assuming a medium effect size, a statistical power of 0.80 and an alpha level of 0.05, a minimum of 60 participants (30 per group) was required. Notably, a previous study identified significant differences in brain metabolite concentrations among three groups: individuals with chronic WAD with neck pain component (*n* = 14), individuals with WAD without neck pain component (*n* = 15) and healthy controls (*n* = 29)^12^.

Thirty individuals with chronic non-specific neck pain were therefore enrolled as part of a clinical trial^13^ and 30 pain-free controls were included from a cross-sectional study on structural brain alterations in individuals with chronic neck pain^14^. All participants were recruited from local hospitals, physical therapy clinics, the universities and/or the community by advertising through flyers, posters and social networks (e.g., Facebook and Instagram). Participants with chronic non-specific neck pain (i.e., unidentified pathoanatomical cause) had neck pain for ≥ 3 months, with an average neck pain intensity over the past week ≥ 35 mm on a 0–100 mm visual analogue scale (VAS). The control group had no history of neck pain or any other persistent pain condition and did not have regular headache or dizziness for at least the past year. All participants were screened for anxiety and depression using the Hospital Anxiety and Depression Scale (HADS)^15^; only those with sub-scores of less than 8 out of 21 on both the anxiety and depression subscales were eligible to be included in the study.

Exclusion criteria for both groups were a history of head and neck injury or surgery; known or suspected vestibular pathology or dizziness caused by underlying pathology in the ear, brain or sensory nerve pathways; neurological or musculoskeletal conditions that could affect the outcomes; metabolic disorders; BMI ≥ 25 kg/m^2^; psychiatric conditions and/or MRI contraindications.

This study was approved by the Ethics Committee of the Faculty of Associated Medical Sciences, Chiang Mai University (No. AMSEC-63EX-101) and was conducted in accordance with the declaration of Helsinki. All participants provided written informed consent prior to participation. They were also asked to refrain from consuming caffeine, alcohol and pain-relief medication on the test day. The reporting of this study conforms with the Strengthening the Reporting of Observational Studies in Epidemiology (STROBE) statement.

### Clinical assessments

#### Self-reported pain outcomes

A general questionnaire was administered to record participants’ demographic and relevant clinical characteristics. For those with neck pain, a 0–100 mm VAS was used to assess pain intensity over the past week, where 0 mm indicated no pain and 100 mm indicated the worst imaginable pain^16^. The VAS has shown to be a reliable tool to measure pain intensity (Intraclass Correlation Coefficient, ICC = 0.97)^17^. Neck disability index (NDI) was used to assess the perceived impact of neck pain on daily activities, with 10 items addressing pain intensity, personal care, lifting, reading, concentration, work, driving, sleep, recreation and headaches. The total score ranges from 0 to 50 and can be expressed as a percentage, with higher scores indicating greater disability^18,19^. The NDI has shown good test-retest reliability in patients with neck pain (ICC = 0.85)^18^. Pain drawings were used to assess pain extent on gender-specific body charts (ventral and dorsal view), regardless of pain intensity and type of pain^20,21^. Drawings were uploaded as a Portable Document Format (PDF) file to the Sketch Your Pain web-based platform (https://syp.spslab.ch), where an automated pain-spot recognition algorithm detected and analyzed the pain drawings. Pain extent was quantified by summing the total pixel count and expressing it as a percentage of the total body chart area (0–100%), excluding any markings beyond the body chart’s borders. The pain drawings have shown excellent test-retest reliability for evaluating pain extent in individuals with neck pain (ICC = 0.92)^22^.

#### Pressure pain thresholds

Pressure pain thresholds (PPTs), defined as the minimum force eliciting a pain response, were measured using a handheld digital pressure algometer (Somedic AB, Farsta, Sweden; 1 cm^2^ probe diameter). The PPTs were assessed over the articular pillars of the C2–3 and C5–6 segments in both the neck pain and control groups. Pressure was increased at a constant rate of 40 kPa/s until participants first perceived a sensation of pain^23^. The PPTs were performed three times at each location and the average values from both sides were used for analysis. One familiarization measure was given to all participants. The intra-rater reliability of PPTs was preliminarily assessed in the study, demonstrating good to excellent reliability (ICC ranging from 0.88 to 0.92).

#### Brain imaging data acquisition and analysis

##### ^1^H-MRS protocol

**Fig. 1:**
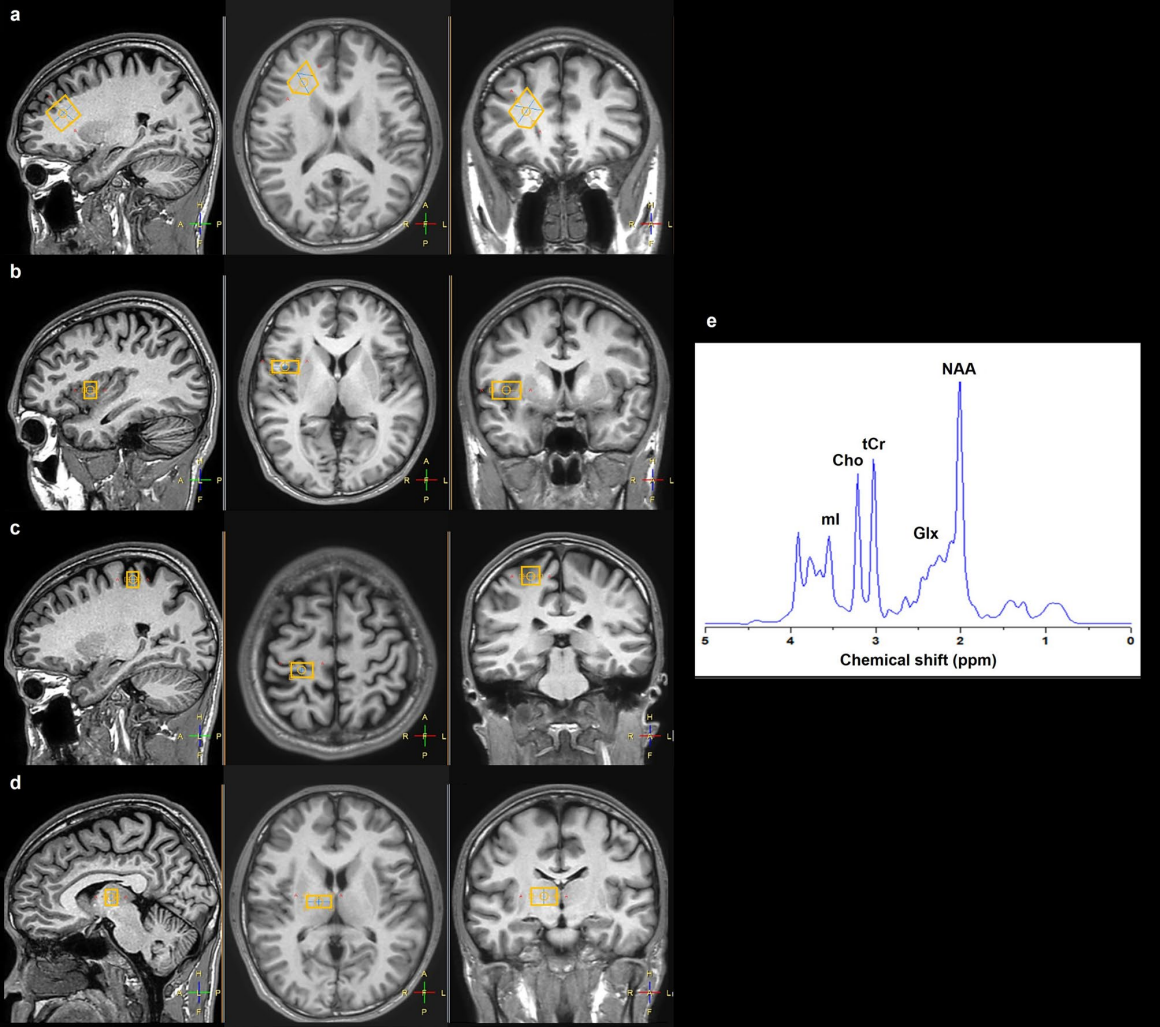
Representative voxel placements in the right hemisphere for (**a**) dorsolateral prefrontal cortex, (**b**) insula, (**c**) primary somatosensory cortex and (**d**) thalamus and (**e**) a corresponding proton magnetic resonance spectroscopy (^1^H-MRS) spectrum. Key metabolites include *N*-acetylaspartate (NAA), total creatine (tCr), choline (Cho), myo-inositol (mI) and the glutamate/glutamine complex (Glx).

Single-voxel^1^H-MRS was performed using a Philips Ingenia 1.5 Tesla MR scanner (Philips Healthcare, Amsterdam, Netherlands) equipped with a SENSE head coil to quantify neurochemical metabolite concentrations in the brain. Prior to the spectroscopic scans, a 3D T1-weighted Turbo Field Echo (TFE) pulse sequence was acquired with the following parameters: repetition time (TR) = 7.4 ms, echo time (TE) = 3.4 ms, field of view (FOV) = 256 × 256 mm^2^, voxel size = 1.0 × 1.0 × 1.0 mm^2^, slice thickness = 1 mm, flip angle = 7° and an acquisition time of 6.10 min. The T1-weighted anatomical images were reconstructed in axial, sagittal and coronal planes to localize the 1 H-MRS voxels in targeted regions based on previous studies^9,12^ and their crucial roles in pain perception and modulation^24^. Voxel sizes were 21 × 17 × 18 mm^3^ in the DLPFC (Fig. 1a), 10 × 22 × 14 mm^3^ in the insular cortex (Fig. 1b), 10 × 15 × 14 mm^3^ in the S1 (Fig. 1c) and 10 × 20 × 13 mm^3^ in the thalamus (Fig. 1d)^25^. Each voxel was carefully positioned by an experienced MRI technician using anatomical landmarks from structural scans to ensure accurate and consistent placement for metabolite extraction while avoiding cerebrospinal fluid and air spaces.

The unsuppressed water signal provided an internal reference for absolute metabolite quantification and was used to correct for eddy current-induced distortions, frequency and phase shifts, and coil sensitivity variations^26^. Water-unsuppressed spectra were acquired from the same voxel prior to the water-suppressed acquisitions, with 16 signal averages to ensure adequate signal quality for referencing and corrections. Water suppression pulses were applied using CHESS to eliminate the dominant water signal, enabling clear visualization of metabolite signals. The point-resolved spectroscopy (PRESS) pulse sequence was used to localize the spectroscopic voxel and acquire metabolite spectra: TR = 2000 ms, TE = 33 ms, number of signal averages = 128, data points = 512 and bandwidth = 1000 Hz. Spectra were visually inspected for quality, focusing on linewidth (Full Width at Half Maximum, FWHM), signal-to-noise ratio (SNR) and absence of artifacts such as baseline distortions, spurious peaks, or lipid contamination. A FWHM value below 8 Hz indicates good magnetic field homogeneity and high spectral resolution^27^. An SNR of 10 or higher is considered sufficient for reliable metabolite quantification, with higher values indicating better spectral quality^27^. Cramer-Rao lower bounds (CRLB) were used to express the precision of metabolite concentration estimates, with values less than 20–30% considered acceptable for reliable quantification^28^. Only spectra meeting these criteria were included for analysis, ensuring accurate and reliable metabolite quantification.

#### Metabolites analysis

Spectral data corresponding to the metabolite peaks were analyzed using the original guideline of the totally automatic robust quantitation in nuclear MR (TARQUIN) software package (version 4.3.10)^29^. Absolute metabolite concentrations were determined by scaling based on the unsuppressed water peak. The spectra data were total creatine (tCr) peak at 3.03 ppm, Cho peak at 3.22 ppm, mI peak at 3.54 ppm, NAA peak at 2.02 ppm and Glx sum peaks at 2.28 and 2.36 ppm^30^ (Fig. 1e).

Spectral normalization was also performed using the peak area of tCr, which is considered relatively stable across different brain regions, physiological conditions and measurement process, making it a reliable internal reference for quantifying other metabolites^31^. Accordingly, the spectra for Cho, mI, NAA and Glx from each participant were normalized to the peak of tCr, expressed as metabolite ratios (i.e., Cho/tCr, mI/tCr, NAA/tCr and Glx/tCr).

### Statistical analysis

Statistical analysis was performed using SPSS, version 22 (IBM Corporation, Armonk, NY). Between-group differences in participant demographic and clinical characteristics were analyzed using independent t-tests and chi-square tests. The normality of the data was assessed using the Shapiro–Wilk test.

Multivariate analysis of variance (MANOVA) was used to analyze differences in absolute metabolite concentrations (i.e., tCr, Cho, mI, NAA and Glx) and metabolite ratios (i.e., Cho/tCr, mI/tCr, NAA/tCr and Glx/tCr) for each brain region. Multiple comparisons were corrected using the Benjamini-Hochberg procedure to control the false discovery rate (FDR), with an adjusted p-value threshold of < 0.05^32^. The FDR correction involved five p-values (tests) for absolute concentrations and four p-values for metabolite ratios. Each hemisphere was analyzed separately. Effect sizes were reported as partial eta squared (η^2^_p_), with values of 0.01 interpreted as small, 0.06 as moderate and 0.14 as large^33^.

Pearson’s correlation coefficient with multiple comparison correction using FDR was performed to evaluate the relationships between metabolite levels (absolute concentrations and ratios; m = 5 and 4 tests, respectively) and pain-related outcomes (adjusted p-values < 0.05)^32^. Correlation strength was categorized as follows: 0.00–0.10 = negligible, 0.10–0.39 = mild, 0.40–0.69 = moderate, 0.70–0.89 = strong and 0.90–1.00 = very strong^34^.

## Results

### Demographic and clinical characteristics

Of the 60 participants, data from one participant with neck pain and four controls were excluded due to baseline folding artifacts that compromised metabolite quantification across all regions. Table 1 presents the demographics and clinical characteristics of the final participants in each group (29 with neck pain and 26 controls). There were no significant differences in age, gender and BMI between the groups (*p* > 0.05). Neither group reported symptoms of anxiety and depression as per the inclusion criteria. Participants with chronic non-specific neck pain experienced moderate neck pain intensity and mild neck disability. PPTs at C2–3 and C5–6 were significantly lower in the neck pain group compared to controls (*p* < 0.001).

**Table 1:** Demographics and clinical characteristics of participants with chronic non-specific neck pain and pain-free controls.

	Neck pain (*n* = 29)	Control (*n* = 26)
**Demographics**		
Gender (n, male/female)	10/19	9/17
Age (years)	31.76 ± 9.52	32.62 ± 10.30
Body mass index (kg/m^2^)	20.97 ± 2.01	21.55 ± 1.98
Side of dominant hand (n, left/right)	1/28	1/25
HADS-anxiety score (0–21)	4.21 ± 1.90	3.46 ± 1.77
HADS-depression score (0–21)	2.62 ± 1.97	2.23 ± 2.01
**Clinical characteristics**		
Side of neck pain (n)		
Unilateral pain (left/right)	1/1	–
Bilateral pain (more painful side, left/right)	16/11	–
Neck pain duration (months)	29.10 ± 29.58	–
Neck pain intensity (VAS, 0–100 mm)	60.00 ± 12.83	–
Neck disability (NDI, 0-100)	21.72 ± 7.65	–
Pain extent (%)	3.55 ± 1.16	–
PPT at C2-3 (kPa)	458.56 ± 140.22	653.73 ± 161.17*
PPT at C5-6 (kPa)	480.14 ± 125.80	682.56 ± 167.54*

Data are presented as mean ± standard deviation unless otherwise indicated. *p < 0.001. HADS, Hospital Anxiety and Depression Scale; VAS, visual analog scale; NDI, Neck Disability Index; PPT, pressure pain threshold.

### MRS analysis

The quality of the spectra across all regions and metabolites enabled accurate quantification of metabolite intensities, supporting robust comparison between groups. For the neck pain group, spectral quality metrics ranged as follows: FWHM from 4.50 to 6.19 Hz, SNR from 10.11 to 28.60 and CRLB from 8.65% to 28.94%. For the control group, FWHM ranged from 4.31 to 6.09 Hz, SNR from 10.10 to 28.18 and CRLB from 7.92% to 28.75%.

#### Absolute metabolite concentrations

**Fig. 2:**
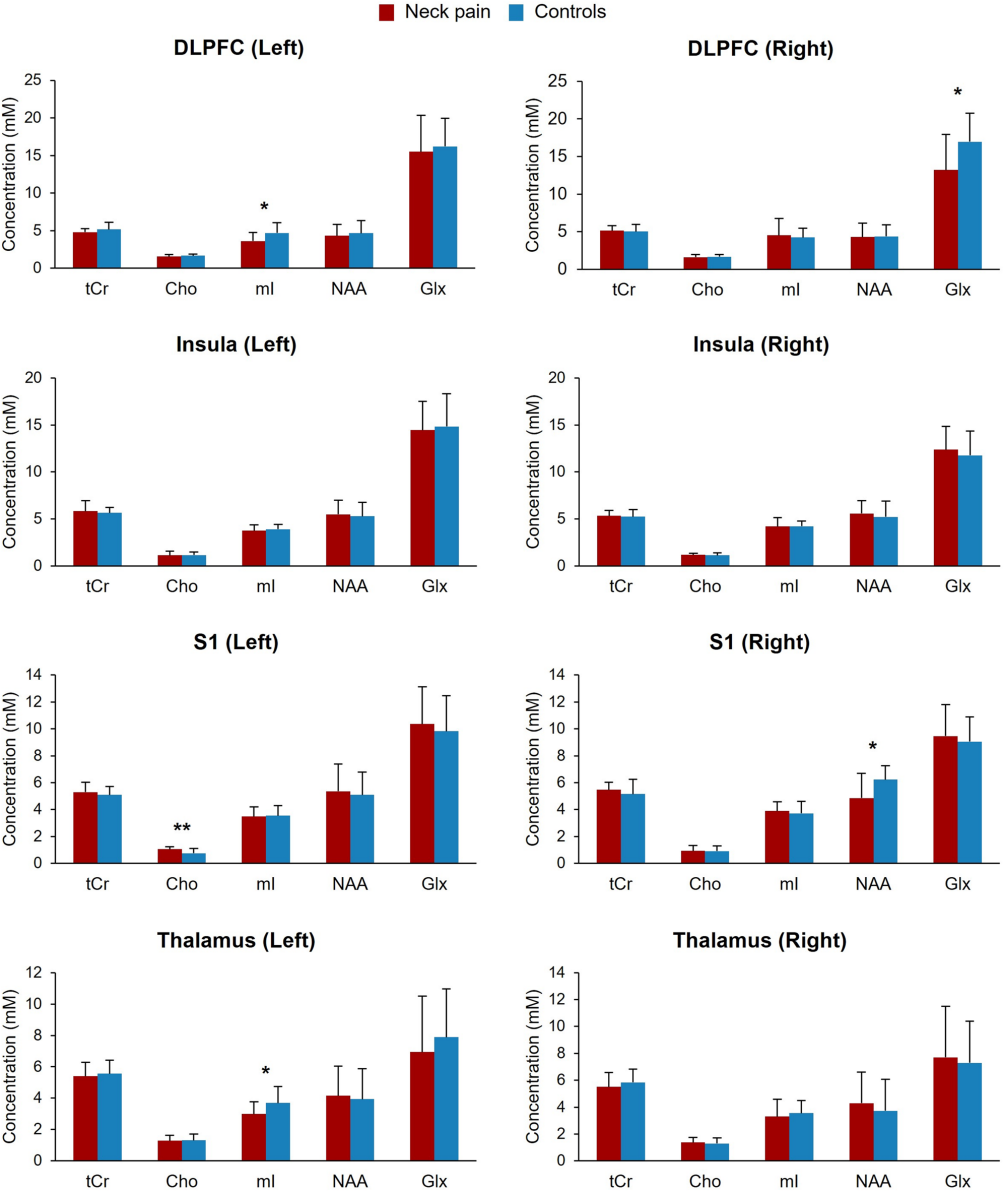
Absolute metabolite concentrations between participants with chronic non-specific neck pain and pain-free controls. Concentrations are presented in millimolar (mM). DLPFC, dorsolateral prefrontal cortex; S1, primary somatosensory cortex; tCr, total creatine; Cho, choline; mI, myo-inositol; NAA, *N*-acetylaspartate; Glx, glutamate/glutamine. *Adjusted p-value < 0.05. **Adjusted p-value < 0.01.

After correction for multiple comparisons, the chronic non-specific neck pain group exhibited decreased mI levels in the left DLPFC and thalamus, Glx level in the right DLPFC and NAA level in the right S1 (adjusted p-values < 0.05, Fig. 2; Table 2). Increased Cho level was observed in the left S1 (adjusted p-value = 0.01, Fig. 2; Table 2).

**Table 2:** Mean difference and 95% CI of absolute metabolite concentrations in participants with chronic non-specific neck pain (*n* = 29) compared to pain-free controls (*n* = 26).

Brain region	Metabolite concentrations	Left hemisphere			Right hemisphere		
		Mean difference (95% CI)	Adjusted *p*-value	η_*p*_^2^	Mean difference (95% CI)	Adjusted *p*-value	η_*p*_^2^
DLPFC	tCr	− 0.36 (− 0.76 to 0.05)	0.20	0.06	0.09 (− 0.37 to 0.55)	0.86	0.00
	Cho	− 0.11 (− 0.25 to 0.03)	0.20	0.05	− 0.05 (− 0.22 to 0.12)	1.00	0.01
	mI	− 1.07 (− 1.76 to − 0.38)	**0.02**	0.16	0.29 (− 0.72 to 1.30)	0.94	0.01
	NAA	− 0.34 (− 1.21 to 0.54)	0.56	0.01	− 0.07 (− 1.03 to 0.89)	0.89	0.00
	Glx	− 0.71 (− 3.10 to 1.67)	0.55	0.01	− 3.75 (− 6.14 to − 1.36)	**0.02**	0.17
Insula	tCr	0.19 (− 0.29 to 0.68)	1.00	0.01	0.09 (− 0.28 to 0.46)	0.77	0.01
	Cho	− 0.03 (− 0.24 to 0.18)	0.79	0.00	0.06 (− 0.05 to 0.18)	1.00	0.02
	mI	− 0.16 (− 0.46 to 0.15)	1.00	0.02	− 0.02 (− 0.44 to 0.41)	0.94	0.00
	NAA	0.21 (− 0.59 to 1.01)	1.00	0.01	0.37 (− 0.46 to 1.20)	0.62	0.02
	Glx	0.39 (− 1.39 to 2.16)	0.83	0.00	0.61 (− 0.75 to 1.97)	0.93	0.02
S1	tCr	0.21 (− 0.18 to 0.60)	0.70	0.02	0.32 (− 0.18 to 0.81)	0.51	0.03
	Cho	0.30 (0.13 to 0.46)	**0.01**	0.22	0.02 (− 0.21 to 0.25)	0.88	0.00
	mI	− 0.06 (− 0.48 to 0.36)	0.77	0.00	0.18 (− 0.28 to 0.64)	0.72	0.01
	NAA	0.26 (− 0.81 to 1.34)	0.78	0.01	− 1.37 (− 2.23 to − 0.51)	**0.01**	0.18
	Glx	0.53 (− 1.02 to 2.07)	0.83	0.01	0.41 (− 0.80 to 1.62)	0.62	0.01
Thalamus	tCr	− 0.16 (− 0.64 to 0.32)	0.85	0.01	− 0.34 (− 0.90 to 0.22)	1.00	0.03
	Cho	− 0.01 (− 0.22 to 0.20)	0.90	0.00	0.09 (− 0.13 to 0.31)	0.70	0.01
	mI	− 0.71 (− 1.23 to − 0.19)	**0.04**	0.13	− 0.24 (− 0.86 to 0.37)	0.54	0.01
	NAA	0.22 (− 0.84 to 1.29)	0.84	0.00	0.57 (− 0.68 to 1.82)	0.92	0.02
	Glx	− 0.94 (− 2.79 to 0.92)	0.79	0.02	0.41 (− 1.48 to 2.30)	0.67	0.00

Data are presented as adjusted mean difference, neck pain vs. control (95% CI, Confidence Interval). DLPFC, dorsolateral prefrontal cortex; S1, primary somatosensory cortex; tCr, total creatine; Cho, choline; mI, myo-inositol; NAA, N-acetylaspartate; Glx, glutamate/glutamine.

#### Metabolite ratios

**Fig. 3:**
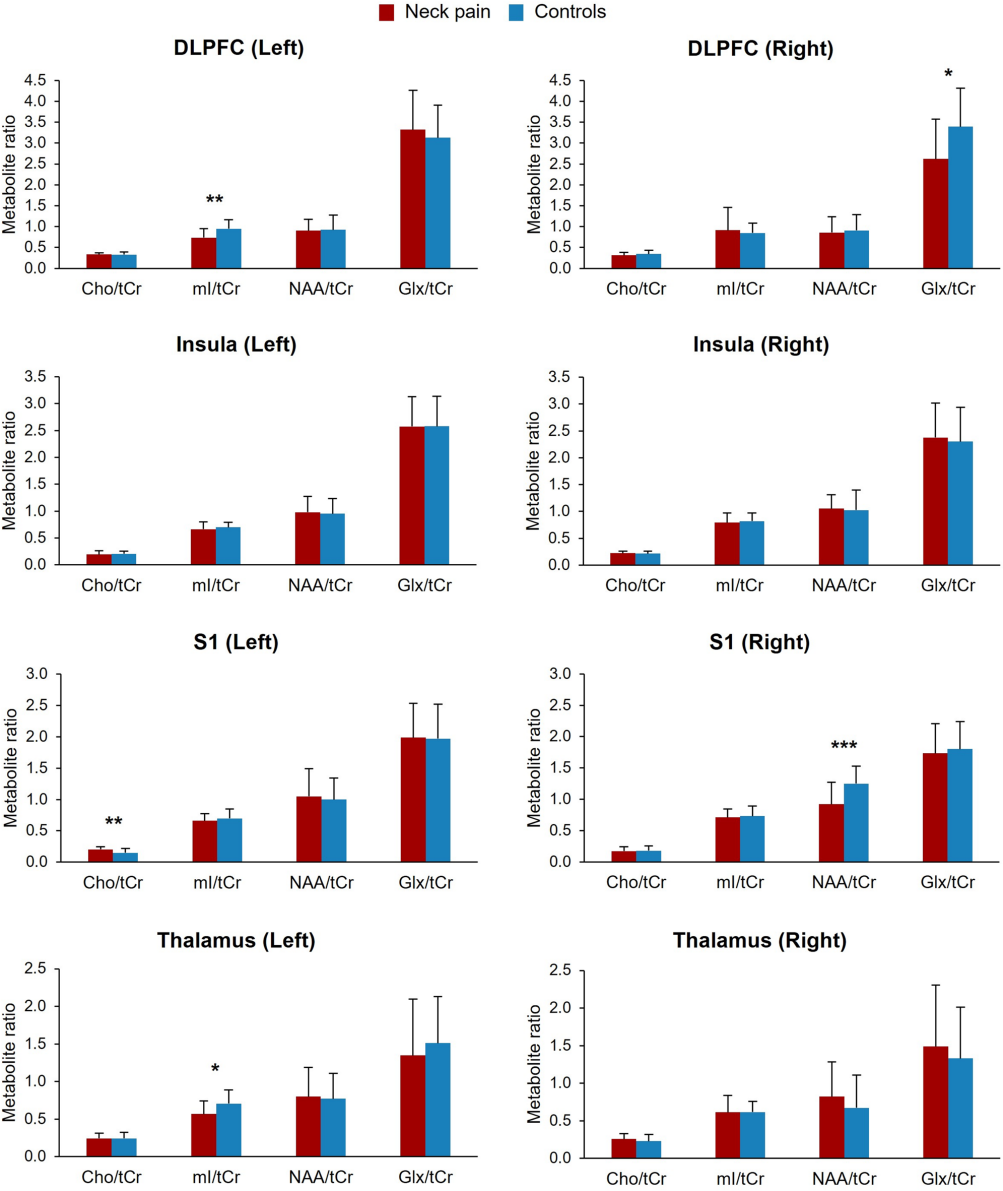
Metabolite ratios between participants with chronic non-specific neck pain and pain-free controls. DLPFC, dorsolateral prefrontal cortex; S1, primary somatosensory cortex; tCr, total creatine; Cho, choline; mI, myo-inositol; NAA, *N*-acetylaspartate; Glx, glutamate/glutamine. *Adjusted p-value < 0.05. **Adjusted p-value < 0.01. ***Adjusted p-value < 0.001.

After correction for multiple comparisons, the chronic non-specific neck pain group showed decreased mI/tCr in the left DLPFC and thalamus, Glx/tCr in the right DLPFC and NAA/tCr in the right S1 (adjusted p-values < 0.05, Fig. 3; Table 3). Increased Cho/tCr was observed for the left S1 (adjusted p-value = 0.01, Fig. 3; Table 3).

**Table 3:** Mean difference and 95% CI of metabolite ratios in participants with chronic non-specific neck pain (*n* = 29) compared to pain-free controls (*n* = 26).

Brain region	Metabolite ratios	Left hemisphere			Right hemisphere		
		Mean difference (95% CI)	Adjusted *p*-value	η_*p*_^2^	Mean difference (95% CI)	Adjusted *p*-value	η_*p*_^2^
DLPFC	Cho/tCr	0.00 (− 0.03 to 0.03)	0.84	0.00	− 0.03 (− 0.07 to 0.02)	0.46	0.03
	mI/tCr	− 0.21 (− 0.33 to − 0.09)	**0.004**	0.20	0.07 (− 0.17 to 0.30)	0.78	0.01
	NAA/tCr	− 0.02 (− 0.20 to 0.16)	1.00	0.00	− 0.05 (− 0.26 to 0.16)	0.63	0.01
	Glx/tCr	0.19 (− 0.31 to 0.69)	0.89	0.01	− 0.77 (− 1.29 to − 0.26)	**0.02**	0.15
Insula	Cho/tCr	− 0.01 (− 0.05 to 0.02)	0.94	0.01	0.01 (− 0.01 to 0.03)	1.00	0.01
	mI/tCr	− 0.03 (− 0.10 to 0.03)	1.00	0.02	− 0.03 (− 0.12 to 0.06)	1.00	0.01
	NAA/tCr	0.03 (− 0.13 to 0.19)	0.96	0.00	0.03 (− 0.15 to 0.20)	0.75	0.00
	Glx/tCr	− 0.00 (− 0.31 to 0.30)	0.98	0.00	0.07 (− 0.27 to 0.42)	0.90	0.00
S1	Cho/tCr	0.05 (0.02 to 0.08)	**0.01**	0.18	− 0.01 (− 0.05 to 0.03)	0.70	0.00
	mI/tCr	− 0.04 (− 0.11 to 0.04)	0.64	0.02	− 0.02 (− 0.10 to 0.06)	0.83	0.01
	NAA/tCr	0.05 (− 0.17 to 0.28)	0.85	0.01	− 0.33 (− 0.51 to − 0.15)	**< 0.001**	0.22
	Glx/tCr	0.02 (− 0.29 to 0.33)	0.92	0.00	− 0.07 (− 0.32 to 0.19)	1.00	0.01
Thalamus	Cho/tCr	0.00 (− 0.04 to 0.04)	0.96	0.00	0.03 (− 0.02 to 0.07)	0.87	0.03
	mI/tCr	− 0.14 (− 0.24 to − 0.04)	**0.03**	0.14	− 0.00 (− 0.11 to 0.10)	0.96	0.00
	NAA/tCr	0.03 (− 0.18 to 0.24)	1.00	0.00	0.15 (− 0.09 to 0.40)	0.44	0.03
	Glx/tCr	− 0.17 (− 0.56 to 0.23)	0.80	0.02	0.16 (− 0.25 to 0.57)	0.59	0.01

Data are presented as adjusted mean difference, neck pain vs. control (95% CI, Confidence Interval). DLPFC, dorsolateral prefrontal cortex; S1, primary somatosensory cortex; tCr, total creatine; Cho, choline; mI, myo-inositol; NAA, N-acetylaspartate; Glx, glutamate/glutamine.

### Correlations between metabolite levels and pain-related outcomes

**Fig. 4:**
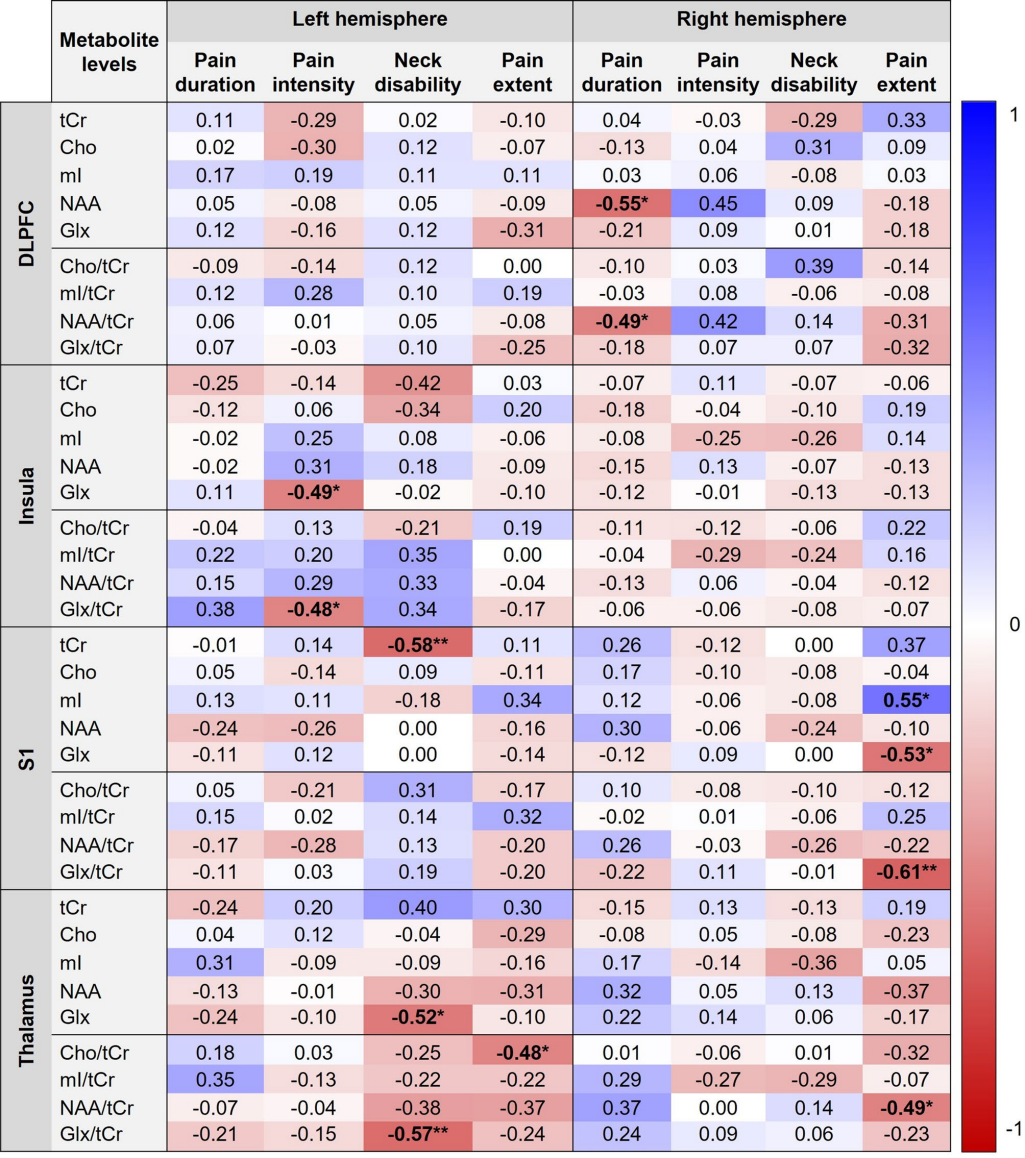
Correlation matrix with values color-coded in blue (positive) or red (negative) between the metabolite levels and self-reported pain outcomes in participants with chronic non-specific neck pain (*n* = 29). *Adjusted p-value < 0.05; **Adjusted p-value < 0.01. DLPFC, dorsolateral prefrontal cortex; S1, primary somatosensory cortex; tCr, total creatine; Cho, choline; mI, myo-inositol; NAA, N-acetylaspartate; Glx, glutamate/glutamine.

Figure 4 presents correlations between the metabolite levels (absolute concentrations and /tCr ratios) and self-reported pain outcomes. After correction for multiple comparisons, several self-reported pain outcomes (i.e., pain duration, pain intensity, neck disability and pain extent) were negatively correlated with specific metabolite concentrations (tCr, mI, NAA and Glx) and ratios (Cho/tCr, NAA/tCr and Glx/tCr) in distinct brain regions (*r* = − 0.48 to − 0.61, adjusted p-values < 0.05). A positive correlation was found between pain extent and mI in the right S1 (*r* = 0.55, adjusted p-value = 0.01).

**Fig. 5:**
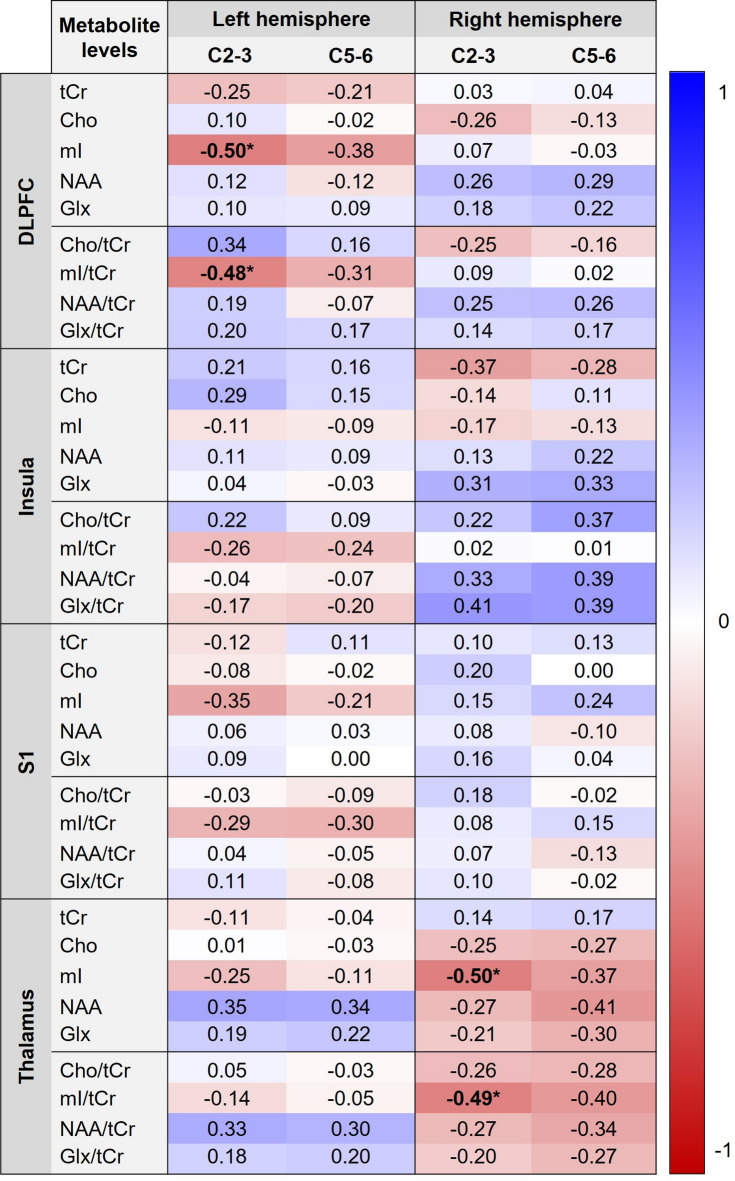
Correlation matrix with values color-coded in blue (positive) or red (negative) between the metabolite levels and pressure pain thresholds measured over the cervical spine in participants with chronic non-specific neck pain (*n* = 29). *Adjusted p-value < 0.05. DLPFC, dorsolateral prefrontal cortex; S1, primary somatosensory cortex; tCr, total creatine; Cho, choline; mI, myo-inositol; NAA, N-acetylaspartate; Glx, glutamate/glutamine.

Figure 5 presents correlations between metabolite levels (absolute concentrations and /tCr ratios) and PPTs measured over the cervical spine. After correction for multiple comparisons, the PPT measured over C2-3 was negatively correlated with mI and mI/tCr in the left DLPFC and right thalamus (*r* = − 0.48 to − 0.50, adjusted p-values < 0.05).

## Discussion

This is the first study to investigate brain metabolite profiles in individuals with chronic non-specific neck pain compared to pain-free controls, providing insights into metabolite alterations and their relationships with pain-related outcomes in this population. Relative to pain-free controls, participants with chronic non-specific neck pain exhibited significantly lower ml in the left DLPFC and thalamus, Glx (glutamate plus glutamine) in the right DLPFC and NAA in the right S1. They also exhibited significantly increased Cho in the left S1. Similar alterations were observed in both absolute concentrations and metabolite ratios relative to tCr (i.e., Cho/tCr, mI/tCr, NAA/tCr and Glx/tCr). In addition, we observed significant correlations between the metabolite levels and pain-related outcomes. These findings may reflect maladaptive neuroplasticity and neuronal–glial alterations within brain networks involved in nociceptive processing and pain regulation in individuals with chronic non-specific neck pain^7,35^. The findings also support the DLPFC, thalamus and S1 as crucial regions in the neurobiology of chronic non-specific neck pain and as potential targets for interventions aimed at restoring normal metabolite levels and improving pain-related outcomes.

Absolute metabolite concentrations represent the actual levels of neurochemicals, whereas metabolite ratios are often used as biomarkers as they can be more sensitive indicators of physiological or pathological changes than individual metabolite levels^31,36^. The reduction in DLPFC and thalamic mI and mI/tCr levels may indicate glial dysfunction or altered osmoregulation as mI acts as an osmolyte in the brain and is essential for maintaining cellular volume and glial cell function^8^. Similarly, the reduction in DLPFC Glx and Glx/tCr levels may reflect impaired glutamatergic signaling, which is important for maintaining excitatory-inhibitory balance and sensory processing in the central nervous system^37^. The DLPFC and thalamus are both integral to pain modulation networks and their functions are often altered in individuals with chronic pain^38,39^. These results are consistent with a previous study on chronic low back pain demonstrating reduced mI levels in the ACC and thalamus^40^.

The findings of reduced NAA and NAA/tCr levels in the S1 are also consistent with a previous study in patients with chronic low back pain^41^. This reduction may indicate altered neuronal mitochondrial metabolism or impaired neuronal health, which can contribute to altered pain processing and perception^42,43^. The S1 is a key region for nociceptive signal processing, where persistent nociceptive input may lead to increased metabolic demand and induce potential mitochondrial dysfunction in S1 neurons, resulting in reduced synthesis of NAA^42,43^. Previous studies reported lower Cho or total Cho levels in pain-related brain regions, including S1, thalamus and DLPFC in individuals with chronic WAD and other musculoskeletal pain conditions^10,12,41^, which may suggest reduced membrane turnover, impaired neurotransmission, smaller cell volume or less myelin synthesis^44,45^. In contrast, our participants with chronic non-specific neck pain exhibited an increase in Cho and Cho/tCr levels in the S1, similar to findings in patients with fibromyalgia^46,47^ and chronic temporomandibular pain^48^. The reasons for these opposing changes are unclear, but increased Cho levels may be associated with neuroinflammation or maladaptive neuroplasticity in response to persistent pain^44,46^. It is also noteworthy that in patients with WAD, one study reported no differences in ratios of metabolite concentrations (i.e., NAA, Cr, Cho, mI and Glx) within the ACC, primary motor cortex and somatosensory cortex (SSC)^49^, whereas another study found lower relative glutamate levels in the ACC and higher tCho levels in the DLPFC compared to controls^12^. The discrepancies between these two studies may be attributed to variations in clinical characteristics of neck pain (e.g., pain intensity and duration) and methodological aspects (e.g., MRS acquisition and brain regions assessed). The insular cortex plays a key role in pain processing, integrating sensory, emotional and cognitive aspects of pain^50^. Our results showed no metabolite changes in the insular cortex in participants with chronic non-specific neck pain, which is inconsistent with previous findings in patients with other chronic pain conditions such as chronic low back pain and fibromyalgia^40,47^. It has been suggested that brain metabolic alterations may result from various factors, including variations in pain characteristics, individual differences, treatment effects and methodological considerations, such as MRS acquisition parameters and spectral quality. The results of this study should therefore be interpreted with caution. Furthermore, brain metabolites may exhibit lateralization influenced by functional specialization of hemispheres and underlying neural circuitry^51^. Nociceptive pathways are known to be lateralized, with pain signals projecting predominantly to the contralateral hemisphere^52^, yet the influence of pain laterality on metabolite patterns remains unclear^53^. In this study, it is also uncertain whether the side of pain location contributed to the findings. Further research specifically addressing pain laterality is warranted.

The results of this study showed moderate correlations between metabolite levels and pain-reported outcomes as well as pressure pain sensitivity over the neck region. Prolonged pain duration, higher pain intensity, greater neck disability and larger pain extent were associated with lower metabolites, including tCr, NAA, NAA/tCr, Glx, Glx/tCr, Cho/tCr in brain regions involved in pain-processing (Fig. 4). Additionally, larger pain extent was associated with higher mI in S1. The variability in the correlations may be due to complex neuroplastic changes and functional reorganization in pain-related brain areas, where altered neuronal activity, glial responses and metabolic shifts interact dynamically during chronic pain states^35^. Some of our findings align with a study on individuals with chronic low back pain, which demonstrated a relationship between decreased NAA levels and prolonged pain duration^41^. However, our results are inconsistent with a previous study in chronic WAD, which demonstrated no relationship between brain metabolites and pain-related outcomes (i.e., duration, pain intensity, neck disability and PPTs measured at the C5 spinous process, median nerve trunks at the elbow and tibialis anterior muscle)^49^. Interestingly, reduced mI and mI/tCr in the DLPFC and thalamus were found to be correlated with lower pressure pain sensitivity measured over C2-3 (Fig. 5). The explanation for this remains unknown, but it may be associated with reduced transmission of nociceptive signals or enhanced descending pain inhibition^38,39^, warranting further investigation. Understanding the relationships between neck pain clinical features and sensitivity and brain metabolite alterations may provide important insights into the central mechanisms underlying chronic non-specific neck pain and allow for the development of targeted intervention strategies.

The study has some limitations that must be acknowledged. Participants in this study were predominantly female and reported only mild to moderate pain and disability, which may limit the generalizability of the findings. However, metabolic changes were still observed, suggesting that physiological alterations occur even at relatively low level of perceived pain intensity. Some potential confounding factors (e.g., medication use and menstrual phase) were not controlled, and certain participants were excluded from the analysis due to baseline folding artifacts. Additionally, tissue segmentation was not performed; therefore, partial-volume effects resulting from heterogeneous tissue composition (gray matter, white matter and CSF), particularly in regions such as the DLPFC and insula, could not be fully eliminated. Metabolite concentrations should be interpreted with caution due to potential partial volume confounds. Furthermore, normalization of voxel positions to MNI space and creation of penetration maps were not performed, which may affect the assessment of spatial consistency across participants and groups. Further research on pain laterality is warranted to determine its impact on brain metabolite patterns. Further studies should also explore the directional relationship between altered metabolite levels and chronic non-specific neck pain and examine whether specific metabolite profiles may serve as prognostic indicators or predictors of treatment response. Randomized controlled trials are also needed to evaluate whether interventions, such as manual therapy and therapeutic exercise, can normalize brain metabolite profiles in individuals with chronic non-specific neck pain.

## Conclusion

Individuals with chronic non-specific neck pain exhibited alterations in brain metabolite levels of Cho, mI, NAA and Glx in the DLPFC, S1 and thalamus compared to pain-free controls. Additionally, some brain metabolites were associated with pain-related outcomes. The findings suggest neurochemical changes in brain regions involved in pain perception and modulation, which may contribute to the experience and persistence of chronic non-specific neck pain.

## Data Availability

The data that support the findings of this study are available from the corresponding author on reasonable request.
